# Analysis of the Influencing Factors of FDM-Supported Positions for the Compressive Strength of Printing Components

**DOI:** 10.3390/ma14144008

**Published:** 2021-07-17

**Authors:** Zhengkai Feng, Heng Wang, Chuanjiang Wang, Xiujuan Sun, Shuai Zhang

**Affiliations:** 1 Robot Research Center, Shandong University of Science and Technology, Qingdao 266590, China; zhengkai3071@foxmail.com (Z.F.); cxjsun@sdust.edu.cn (X.S.); 15069317408@163.com (S.Z.); 2 Qingdao Zhong Jiate Electric Co., Ltd., Qingdao 266000, China

**Keywords:** 3D printing, supporting method, compression, compressive strength comparison, failure form

## Abstract

Fused deposition modeling (FDM) has the advantage of being able to process complex workpieces with relatively simple operations. However, when processing complex components in a suspended state, it is necessary to add support parts to be processed and formed, which indicates an excessive dependence on support. The stress intensity of the supported positions of the printing components can be modified by changing the supporting model of the parts, their density, and their distance in relation to the Z direction in the FDM printing settings. The focus of the present work was to study the influences of these three modified factors on the stress intensity of the supporting position of the printing components. In this study, 99 sets of compression tests were carried out using a position of an FDM-supported part, and the experimental results were observed and analyzed with a 3D topographic imager. A reference experiment on the anti-pressure abilities of the printing components without support was also conducted. The experimental results clarify how the above factors can affect the anti-pressure abilities of the supporting positions of the printing components. According to the results, when the supporting density is 30% and the supporting distance in the Z direction is Z = 0.14, the compressive strength of the printing component is lowest. When the supporting density of the printing component is ≤30% and the supporting distance in the Z direction is Z ≥ 0.10, the compressive strength of printing without support is greater than that of the linear support model. Under the same conditions, the grid-support method offers the highest compressive strength.

## 1. Introduction

3D printing technology was inspired by the concept of photoengraving developed by William Francois, an artist from the 19th century, and then Blanther laid the foundation for it by studying geographical terrain maps [1]. Subsequently, the STL (Stereo lithography) file format became one of the standards in CAD/CAM (computer-aided design/computer-aided manufacturing) [2]. Presently, various industries and companies extensively use 3D printing technology to manufacture complex products [3]. Additive manufacturing (AM) is one of the most promising techniques in component manufacturing [4,5,6,7,8,9]. In this field, fused deposition modeling (FDM) [10] composite filament technology [11,12] has gained extensive attention and is widely used in modern medical and intelligent manufacturing and in the aerospace industry, due to its advantages in manufacturing complex components alongside its simplicity and convenient operation [13,14,15,16].

The process of FDM itself is relatively complicated. FDM requires different target components with different strengths and surface qualities, entailing fine adjustments of the various printing parameters and interactions between the parameters to print the expected target components [17,18,19,20,21,22]. Sood et al. studied the influences of the air gap, grid width, and grating angle on the anti-pressure abilities of printing components [23]. Chacón et al. studied the influences of construction direction, layer thickness, and feed rate on the mechanical properties of polylactic acid (PLA) samples [24]. In addition, fluctuations of the printing temperature can cause separation between the layers, which, in turn, can affect the surface quality and mechanical properties of the printout [25,26,27]. Recently, Marchment, Zheng, and Krystek et al. studied the influence of extending the time and changing the printing path and position on the mechanical properties of other materials, including 3D printed concrete, polyether-ether-ketone/hydroxyapatite composites, and metal prints [28,29,30]. These analyses provided ideas for further research on the mechanical properties of PLA printing components. Suteja, Khawaja, and Mustakangas et al. explored the influence of the internal factors of printed components on compressive strength by studying filling patterns and filling density [31,32,33]. The construction direction can also affect the mechanical strength of FDM components [34]. The fiber directions of the components printed in different construction directions also vary, as do the different supported parts of the printing components, resulting in different stress intensity directions for the printing components, which may cause excessive dependence on the construction direction, similar to that of composite laminated boards [35,36,37]. When printing components, dust on the cardboard can also increase the compressive strength [38]. In the most basic Cura slice software [39], the placement of complex printing components in different directions can also produce variations in the local intensity of the printing components due to the different supporting positions used. However, few published studies have addressed these issues. Analyzing the structural strength of the supported positions of basic reinforced fiber 3D printing components is of special importance in manufacturing [11,40,41,42].

In light of the above analysis, in the present study we investigated the influence of support positions on the compressive strength of printing components. The influence of various FDM support modes, including the influence of support density and the distance from the support to the model in the Z direction, on the compressive properties of the supporting parts of the printing components was analyzed for PLA materials. The experimental components were printed with PLA materials, and the compressive strength of the printed components with different support models, different support densities, and different support distances in the Z direction was studied by compression testing. A total of two series of tests were performed in the test programs. The first test series was used to compare the grid and linear support models of the support part under four support densities (30%, 50%, 70%, and 90%). The second test series was used to compare the grid and linear support models of the support part under four support distances (0.08, 0.10, 0.12, and 0.14 mm). The experimental results were then measured with a 3D topographic imager (Zeta-20). Clearly, the compressive strength of the printing component relates to the undersurface roughness and the adjacent grids. The failure forms of printing components were also analyzed in this study.

## 2. Experimental Methods

### 2.1. Preparation of the 3D Printer, Materials, and Samples

This study mainly investigated the influences of different support models, including the influence of support density and the distance from the support to the model in the Z direction, on the compressive strength of the supporting parts of the printing components. The materials used in this experiment were composed of 1.75 mm PLA (JG MARKER, Shenzhen, China), which uses fermented crops as raw materials with fine cohesiveness, fluidity, degradability, tensile strength, and biocompatibility. The products printed by PLA are environmentally friendly, have high hardness, are of low cost, and offer energy savings. The printing temperature for the material is generally between 190 and 210 °C. Therefore, PLA is one of the best raw materials for 3D printing.

**Figure 1:**
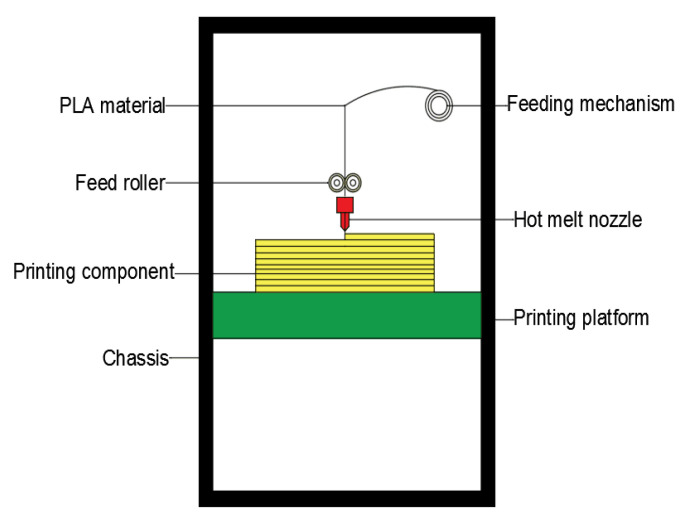
DF-G450 working principle diagram.

To prepare the sample printing, we used a DF-G450 printer (DUFEN, Zhuhai, China), the operating principles of which are shown in Figure 1. The printer had overall dimensions of 1400 mm × 950 mm × 1840 mm and was capable of printing products with dimensions of 400 mm × 500 mm × 600 mm. The experimental sample printing used a jet nozzle with a diameter of 0.8 mm to match the disk-like PLA material, which had a diameter of 1.75 mm.

**Figure 2:**
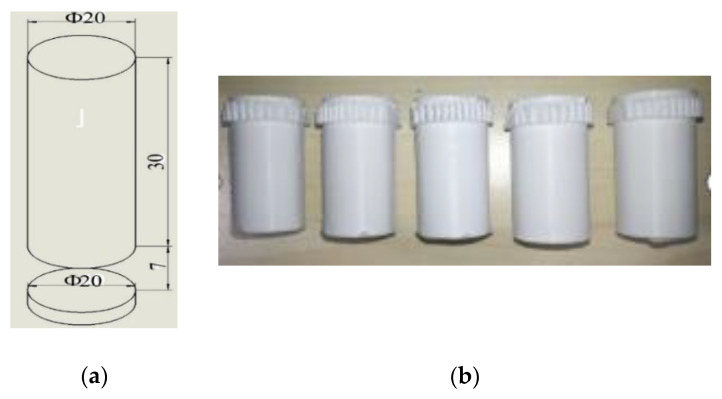
Experimental sample: (**a**) test-piece 3D model diagram; (**b**) photograph of some of the test pieces.

The specific 3D model and the dimensions of the sample are shown in Figure 2a. The pedestal was a column of 20 mm × 2 mm, the support length was 7 mm, and the supporting component was a cylinder of 20 mm × 30 mm. The actual printed model is illustrated in Figure 2b. In keeping with the design parameters of the printing component, the experimental components with the two supporting models included four supporting distances (0.08, 0.10, 0.12, and 0.14 mm) and four supporting densities (30%, 50%, 70%, and 90%). When the supporting density of the supporting part is lower, the surface of the printing component becomes uneven, and the wire-drawing phenomenon becomes more serious. Under the same supporting density, the supporting distance of the printing component increases, the drawing phenomenon gradually decreases, and the surface of the filling pattern becomes flat and smooth. When the component is printed under grid-support conditions, the result is smoother than the linear support surface under the same printing conditions, and the filling pattern is clearer.

### 2.2. Technological Parameter Setting

This experiment studied the influences of different support models, support densities, and distances from the support to the model in the Z direction on the compressive strength of the supporting parts of the components. The support densities and distances are shown in Table 1. However, the mechanical properties of the components manufactured by FDM are also affected by other technological parameters, such as layer thickness, as shown in Table 1. Therefore, it was necessary to set these other parameters before printing to ensure that the components prepared using 1.75 mm PLA materials with the DF-G450 printer could achieve optimal mechanical properties. However, an increase in support density also increases the cross-sectional area, which can affect stress measurements. To meet the experimental requirements, the porosity of the material printed with a 0.2 mm layer thickness was 0.14%, as smaller porosity can reduce the influence of the cross-sectional area on the experiment [43]. In small cross-sectional parts, the nominal infill has less of an effect on the resulting specimens [44]. Moreover, the support density should not be too large (100%) or too small (10% to 20%). A comparison between the four support densities of 30%, 50%, 70%, and 90% was performed to further reduce the influence of the cross-sectional area on the experiment [44]. The other technological parameters were set according to the optimal values recommended by the equipment manufacturer, combining past service experience and practical industrial applications.

### 2.3. Experimental Facility

**Figure 3:**
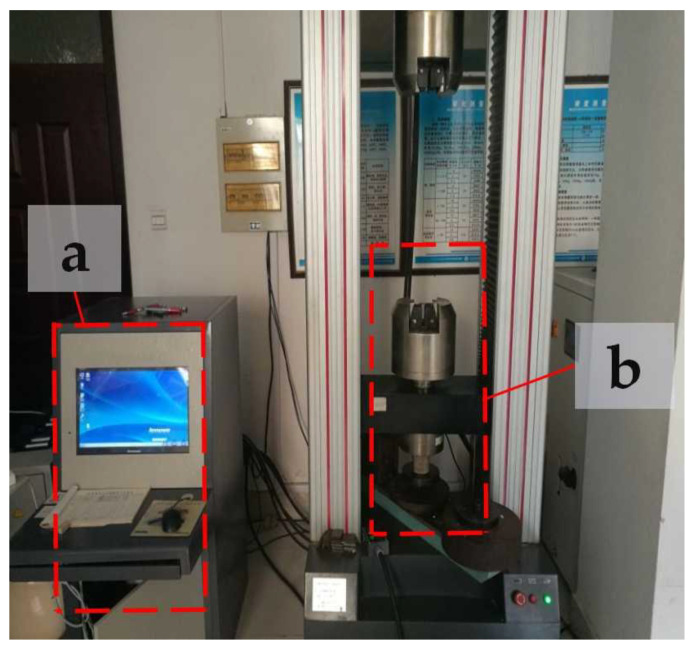
Microcomputer-controlled electronic universal testing machine: (**a**) electronic universal testing machine console; (**b**) electronic universal experiment machine test bench.

**Figure 4:**
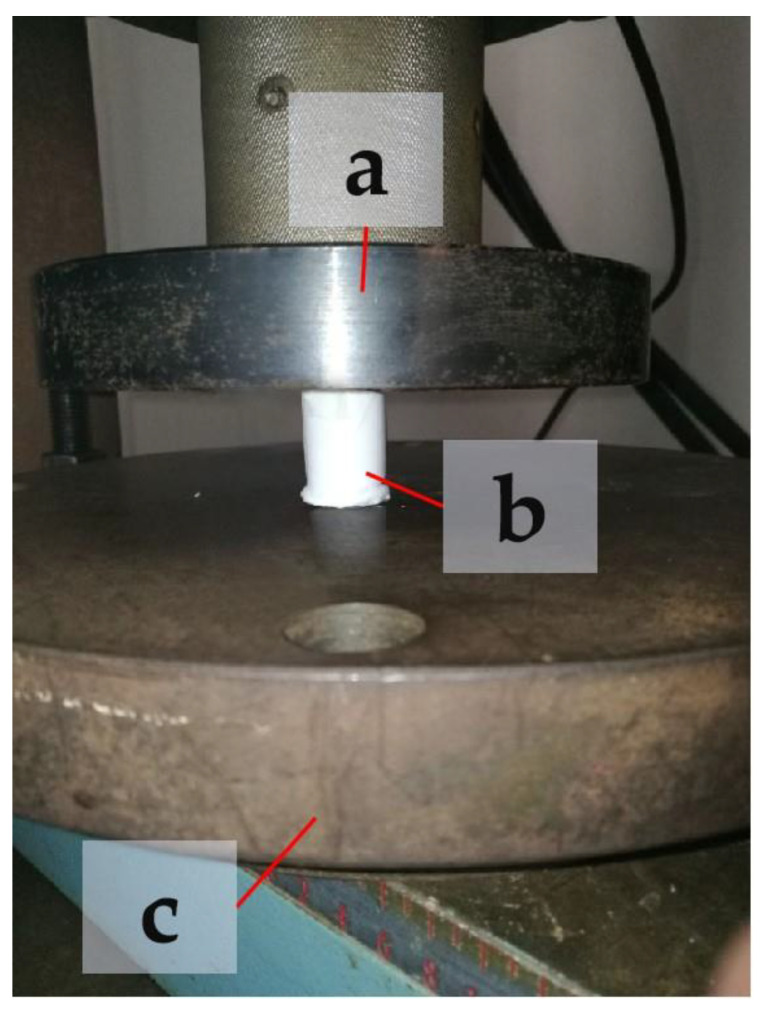
Compression head: (**a**) upper plate; (**b**) experimental component; (**c**) lower plate.

**Figure 5:**
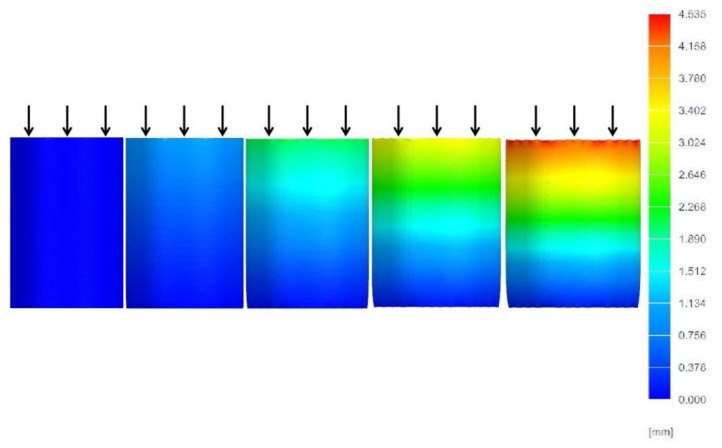
Compression deformation simulation diagram of the print component without support.

To reduce the influence of fluctuations in the support parameters on the experimental results, three samples in each group were printed using the same supporting conditions for each supporting model. As a reference, a group of three samples was also printed without support. In total, our experiment included 37 groups and 99 experimental samples. Since PLA is a moisture-absorbing material under normal conditions, in order to reduce the influence of errors on the experimental results caused by the properties of the material, the experimental samples were printed with vacuum-packed PLA printing materials in a moisture-absorbing feed box. All the tests were conducted at a temperature of 23 ± 2 °C and relative humidity of 50 ± 5%. The average compressive strength values of the experimental samples in each group were collected as the test results. For the strength test, we used a microcomputer-controlled electronic universal testing machine (WDW-200E) (ZONGCHI, Jinan, China), as shown in Figure 3. The flat-head compression rate was set to 2 mm/min, and the full-scale load scope was set at 200 KN. The process of extruding experimental samples is shown in Figure 4. To reduce the influence of the end effects on the experimental results, a small amount of lubricant oil was applied to the end surface of the printing component. During the compression process, the sample with the largest support distance in the Z direction expanded laterally more than the other samples. Under the same support distance, with an increase in support density, the change in the support position decreased. When the supporting density of the printing component = 30% and the supporting distance in the Z direction was Z = 0.10, the changes in the components printed without support and those printed using the linear support model were largely the same. The results show that the components printed with grid support are stronger than those printed with linear support when using the same parameters. Figure 5 shows the compression deformation simulation diagram of the print component without support.

## 3. Results and Discussion

### 3.1. Experimental Analysis of Linear Support

Table 2 shows the average compressive strength of the experimental samples with different support densities and distances from the support to the model in the Z direction under conditions of linear support. The average compressive strength of the printing component test without support was 20.2 MPa.

**Figure 6:**
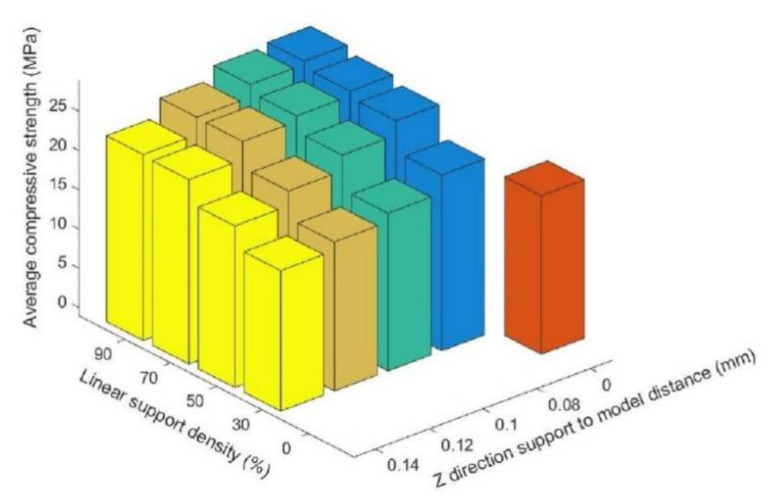
Relationship between density, Z direction support-to-model distance, and average compressive strength under linear support.

Table 2 compares the average compressive strength of the supporting parts of the printing components with linear support and that of the printing components without support. Clearly, when the printing condition of the supporting part of the printing component satisfies Z ≥ 0.10, and the linear packing density is ≤30%, the compressive strength of the printing component without support will be larger than that of the supporting part of the printing component with linear support. Figure 6 shows the relationship between density, Z direction support-to-model distance, and average compressive strength under the linear support model.

**Figure 7:**
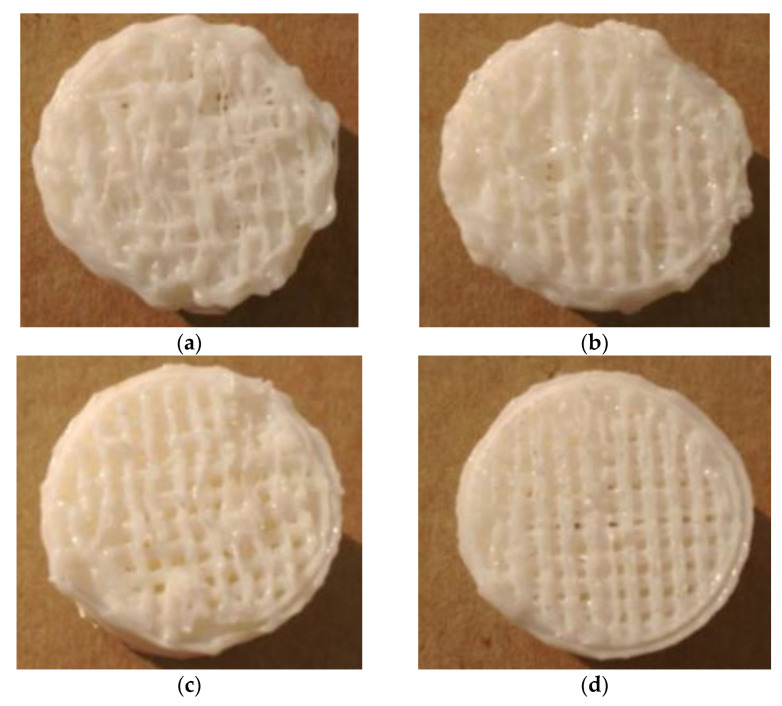
Images of the bottoms of the linear support experimental parts removed under different linear support densities: (**a**) 30% linear support density (bottom view); (**b**) 50% linear support density (bottom view); (**c**) 70% linear support density (bottom view); (**d**) 90% linear support density (bottom view).

Figure 6 shows that, under the condition of linear support, with the same distance from the support to the model in the Z direction, the compressive strength of the supported part of the printing components increased with an increase in the linear support density, but the rate of increase decreased with an increase in the support density. Figure 7 shows the surfaces of the Z = 0.08 components for the supported part of the printing components after the support was removed (where the support densities were a = 30%; b = 50%; c = 70%; and d = 90%). After the support was removed, the undersurface became smoother, and the support density increased.

As the grid is laminated by liquid PLA, which is melted from solid filiform PLA via a heating nozzle, when printing the underneath surface of the supporting part of the component, the liquid silk ejected from the nozzle quickly adheres to the solidified support at the top. This newly ejected high-temperature liquid silk can melt quickly on the upper surface of the support and cause local secondary melting. This melting can cause the fine silk to cool on the upper layer and form an asymmetrical temperature gradient along the direction of sedimentation. The thermal stress caused in this way can then deform the component. This secondary melting can distribute liquid silk onto the lower surface of the supporting part of the printing component drawbench because the support gap is not sufficient. During this process, clashes between neighboring grids can result in overlaps and form a zero-air gap grid, which can make the printed surface uneven, with fractures and pits. These fractures and pits can seriously affect the compressive strength of the supporting parts of the printing components. The smaller the support density is, the larger the support gap and the more serious the drawbench will become, as shown in Figure 7a.

**Figure 8:**
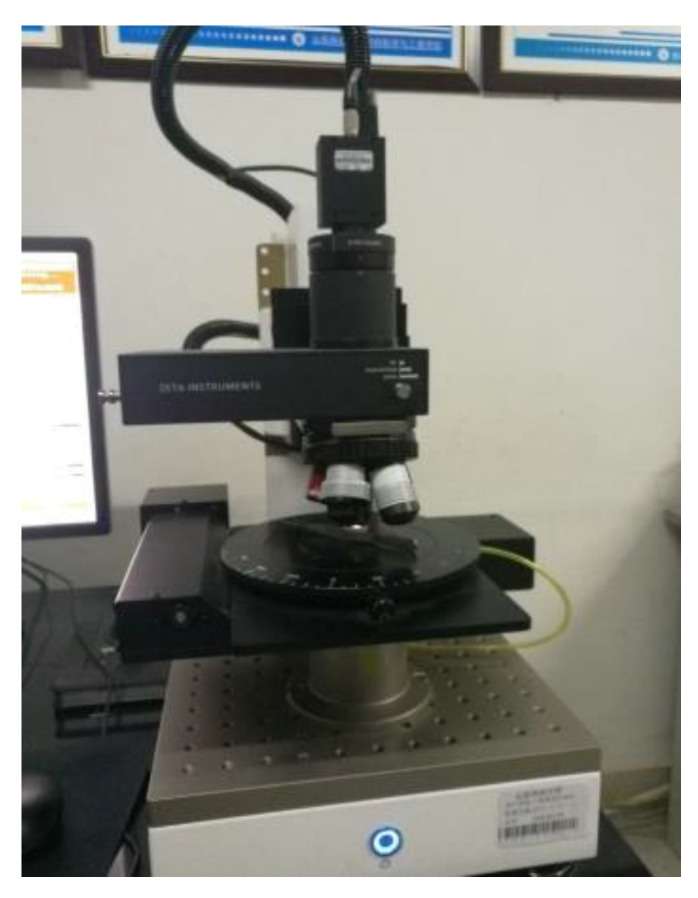
Three-dimensional topographic imager (Zeta-20).

**Figure 9:**
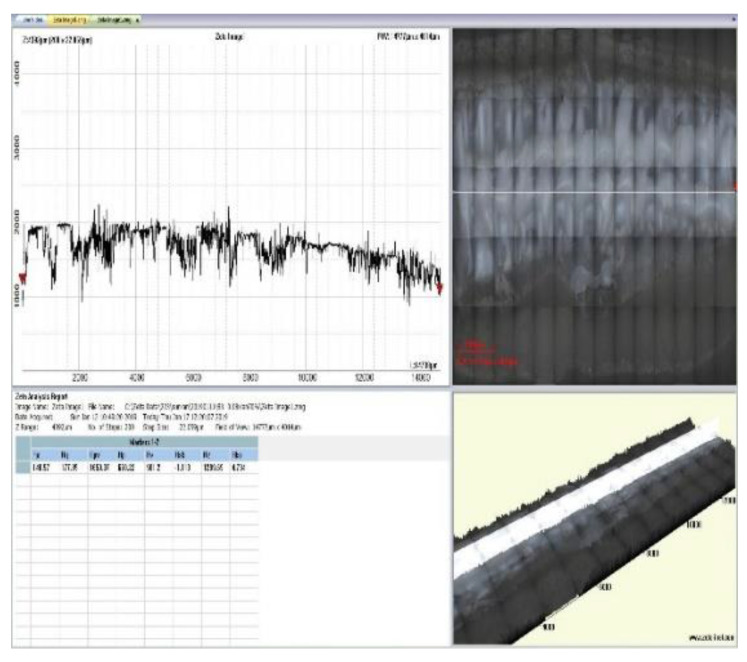
Zeta-20 test display interface.

Based on the above analysis of the experimental results, the compressive strength of the supporting parts of the printing components relates to the undersurface roughness and the adjacent grids. To analyze the surface roughness, two neighboring grids at the same arbitrary locations as the supporting parts of the four printing components with linear support densities of 70% and 90% and Z direction distances of Z = 0.08 and Z = 0.14 were measured with a 3D topographic imager (Zeta-20) (ZETA, Ca, USA), as shown in Figure 8. The overall widths of the two neighboring grids and the gap generated between the adjacent grids are shown in Figure 9.

**Figure 10:**
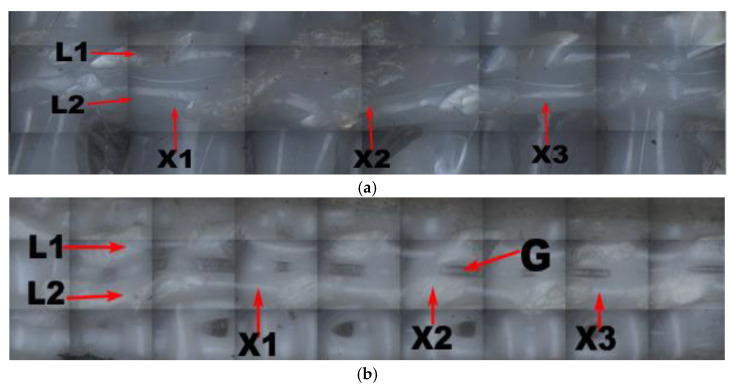

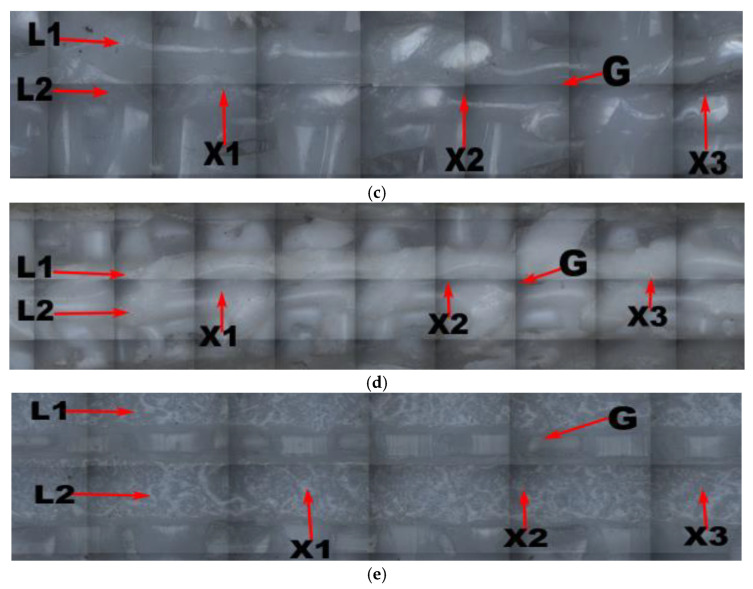
Two adjacent raster images of printed components under different conditions: (**a**) 70% linear support density and grating of z = 0.08; (**b**) 90% linear support density and grating of z = 0.08; (**c**) 70% linear support density and grating of z = 0.14; (**d**) 90% linear support density and grating of z = 0.14; (**e**) lower surface of unsupported printing member.

Three-dimensional images of two neighboring grids of the supported part of each of the four printing components with linear support and of the adjacent grid without support were observed using the Zeta-20 imager, as shown in Figure 10 (linear support density and distance in the Z direction from the support to the surface of the supported part: a = 70%, z = 0.08; b = 90%, z = 0.08; c = 70%, z = 0.14; d = 90%, z = 0.14; e = 0%, z = 0). Here, G is the gap formed between two adjacent grids; L1 and L2 are two neighboring grids whose grid roughness, $R_{a1}$ and $R_{a2}$, was measured to obtain the average grid roughness $\bar{R_{a}}$; and X1, X2, and X3 represent the total width formed after printing the measured adjacent grids, the combination of which makes it possible to obtain the average width $\bar{X}$ of the neighboring grids.

Figure 10 shows that, with a fixed distance from the linear support to the undersurface of the supporting part in the Z direction, the gap between two neighboring grids increased with an increase in linear support density, but the overall relative size and gaps between the neighboring grids were unevenly distributed. In Figure 10a, there is almost no gap visible between the two neighboring grids, indicating a zero-air gap grid, which is beneficial to the formation of intense bonding. Though intense bonding can lead to slight unevenness in the local surface due to overlapping of the grid when the component is compressed, the formation of intense bonding can enhance the compressive performance of the supported components. Furthermore, Figure 10b shows that with an increase in the support density, the surface of the supported neighboring grid became more even, and small air gaps began to form between the neighboring grids, thereby facilitating firm bonding between the two grids and improving the compressive strength. However, small air gaps can inhibit heat dissipation, which can increase the possibility of stress accumulation. Due to the slight enlargement of the air gap between the grids and the surface roughness, and with the distance from the linear support to the undersurface of the supporting part in the Z direction unchanged, the increased rate of the compressive strength of the printing components slowed with an increase in the linear support density. In Figure 10e, the size and distribution of the gaps between the two neighboring grids on the undersurface without support are even, and the air gap between the neighboring grids is large.

Table 3 shows that, when the distance from the linear support to the undersurface of the supported part remained unchanged, with an increase in the linear support density, the average surface roughness $\bar{R_{a}}$ of the two adjacent grids decreased. It can thus be inferred that the undersurface of the supported part of the printing component was smoother. On the other hand, with an increase in the support density, the contact points between the undersurface grating of the supporting part and the upper surface grating of the linear support increased, as did the PLA on the upper surface of the secondary melting support, thereby increasing the average total printing width of the adjacent grating $\bar{X}$. Moreover, the average surface roughness $\bar{R_{a}}$ of the supporting component was low, and the undersurface was smooth. As the direct contact between the undersurface of the printing component without the support and the printing platform did not undergo secondary fusion, the undersurface of the printing component was smooth, and the average total printing width $\bar{X}$ of the adjacent grids was larger than that of the adjacent grids on the undersurface of the supported part of the supporting component.

Further, as the distance from the support to the undersurface of the supported part of the printing component increased in the Z direction, the contact area of the solid silk PLA ejected by the hot melting nozzle with linear support decreased and the secondary melting of the linear support grids weakened. Thus, with the linear support density unchanged, the average surface roughness $\bar{R_{a}}$ of two adjacent grids would decrease with an increase in the distance from the linear support to the undersurface of the supporting part in the Z direction, while the undersurface of the supported part would remain relatively smooth after the linear support is removed. Moreover, the average total printing width $\bar{X}$ of the adjacent grids decreases.

### 3.2. Experimental Analysis of Grid Support

Table 4 shows the average compressive strength of the experimental samples with different support densities and distances from the support to the model in the Z direction under conditions of grid support.

**Figure 11:**
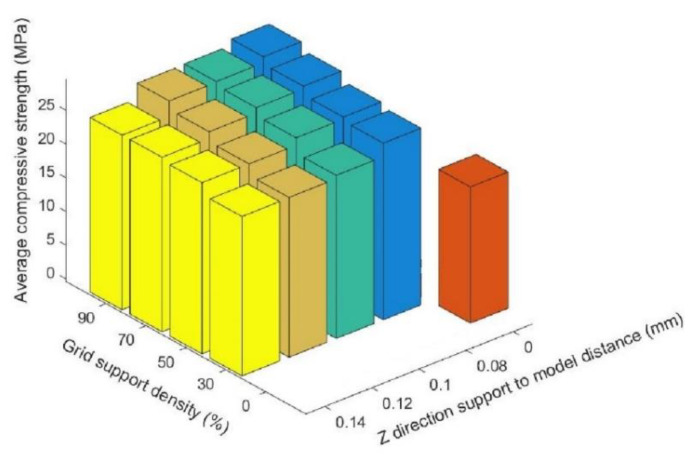
Relationship between density and Z direction support-to-model distance—average compressive strength under grid support.

Table 4 compares the average compressive strength values of the supporting parts of the printing components with grid support with those of the printing components without support. Likewise, when the supported part of the printing component under the condition of grid support satisfies Z ≥ 0.08 and the linear packing density is ≤30%, the compressive strength of the printing component without support is smaller than that of the supporting part of the printing component with grid support. Figure 11 shows the relationship between density, Z direction support-to-model distance, and average compressive strength under the grid-support model.

**Figure 12:**
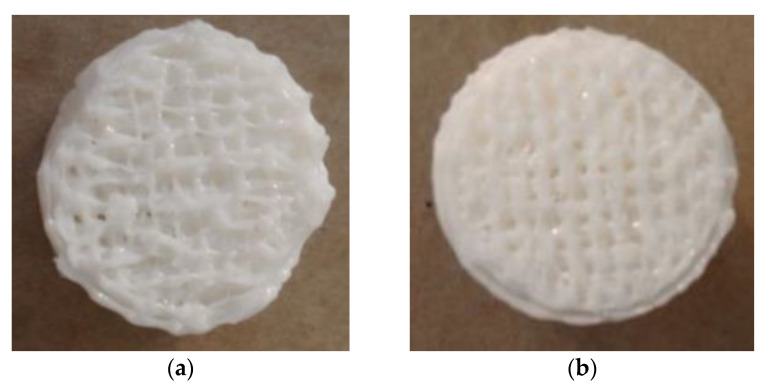

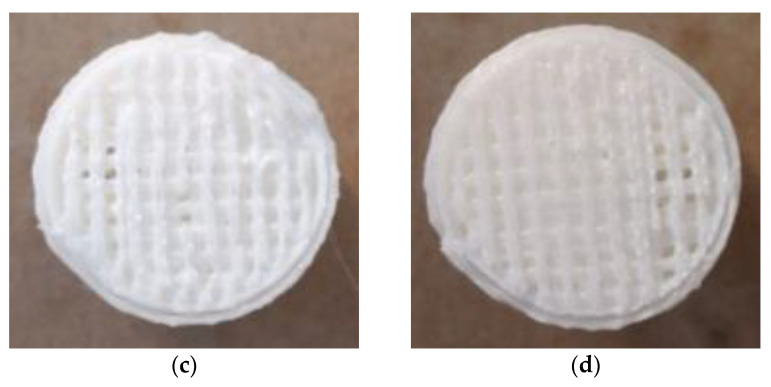
Images of the bottoms of the experimental grid-support parts removed under different grid support densities: (**a**) 30% grid support density (bottom view); (**b**) 50% grid support density (bottom view); (**c**) 70% grid support density (bottom view); (**d**) 90% grid support density (bottom view).

Figure 11 shows that, under conditions of grid support, when the distance from the support to the model in the Z direction was the same, the compressive strength of the supporting parts of the printing components increased with an increase in the support density, but the increase rate slowed with an increase in the support density. Figure 12 shows the surfaces of the Z = 0.08 components for which the supported parts had their supports removed (where the support densities were a = 30%; b = 50%; c = 70%; d = 90%). After the grid support was removed, with an increase in the support density, the drawbench on the undersurface of the supported part of the printing component decreased, and the support surface became smoother, with no large-scale pits resulting from the drawbench.

As shown in Figure 7 and Figure 12, with the same distance from the support to the model in the Z direction and the same support density, when comparing the surfaces of the supporting parts of the printing components with the grid supports and the surfaces of the supporting parts of the printing components with linear support, the grid shape of the supporting surfaces of the printing components with grid support were more regular and evenly distributed.

**Figure 13:**
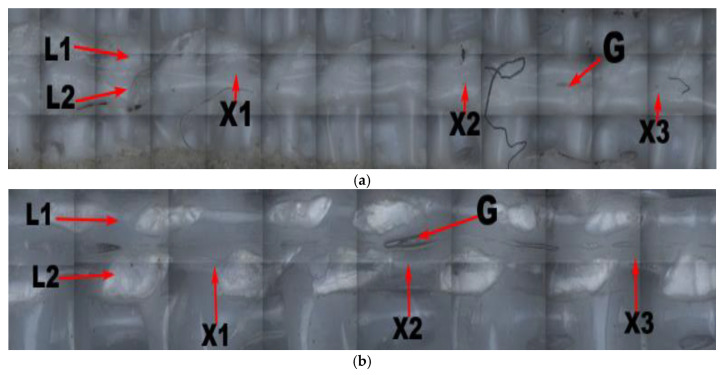

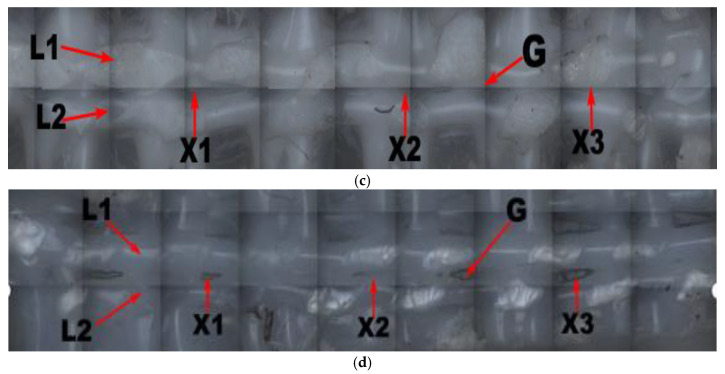
Two adjacent raster images of printed components under different conditions: (**a**) 70% grid support density and grating of z = 0.08; (**b**) 90% grid support density and grating of z = 0.08; (**c**) 70% grid support density and grating of z = 0.14; (**d**) 90% grid support density and grating of z = 0.14.

Three-dimensional images of two neighboring grids of the supported part of each of the four printing components with linear support and the neighboring grid without support (under the condition of grid support) are shown in Figure 13 (grid support density and the distance in the Z direction from the support to the surface of the supported part: a = 70%, z = 0.08; b = 90%, z = 0.08; c = 70%, z = 0.14; d = 90%, z = 0.14; e = 0%, z = 0). Here, G is the gap formed between two neighboring grids, and L1 and L2 are the two neighboring grids; the grid roughness, $R_{a1}$ and $R_{a2}$, was measured to obtain the average grid roughness $\bar{R_{a}}$; and X1, X2, and X3 are the total widths formed after printing the measured neighboring grids, the combination of which makes it possible to obtain the average width $\bar{X}$ of the neighboring grids.

Figure 13 shows that when the distance from the grid support to the undersurface of the supported part remained unchanged in the Z direction, with an increase in grid-support density, the gap between two neighboring grids increased. However, the relative sizes and distributions of the gaps were uneven. Figure 13a shows that there were almost no gaps between the two neighboring grids, and zero-air gap grids were formed in most locations.

Figure 13a shows that, with an increase in the support density, the gap between the two grids at the undersurface of the grid support component was nearly smooth, with a zero-air gap grid. Moreover, Figure 13b shows that, with an increase in support density, the grids on the supporting surface were more uniform, and small air gaps began to form between adjacent grids on the undersurface of the supported part of the printing components with grid support. Due to the slight enlargement of the air gap between the grids and the material’s surface roughness, when the distance from the grid support to the undersurface of the supporting part in the Z direction remained unchanged, the increased rate of the compressive strength of the printing components slowed with an increase in grid support density.

Based on Table 5, when Z = 0.08, with an increase in grid support density, the average surface roughness $\bar{R_{a}}$ of two adjacent grids decreased from 178 to 139.5 μm. Therefore, with an increase in grid support density, the undersurface of the supported part of the printing component became smoother. At the same time, with an increase in support density, the PLA on the upper surface of the secondary melting support increased, thereby increasing the average total printing width of the adjacent grids $\bar{X}$. Moreover, the average surface roughness $\bar{R_{a}}$ of the component without support was low, and the undersurface was smooth. As the direct contact between the undersurface of the printing component without the support and the printing platform cannot undergo secondary fusion, the average total printing width $\bar{X}$ of the adjacent grids was larger than that of the adjacent grids on the undersurface of the supported part of the supporting component.

Moreover, because the distance from the support to the undersurface of the supported part of the printing component increased in the Z direction, the contact area of the solid silk PLA produced by the hot melting nozzle with linear support decreased, and secondary melting of the grating support grids weakened. Thus, with the grid support density unchanged, the average surface roughness $\bar{R_{a}}$ of two adjacent gratings decreased with an increase in the distance from the grid support to the undersurface of the supporting part in the Z direction, while the undersurface of the supported part remained relatively smooth after the linear support was removed. Moreover, the average total printing width $\bar{X}$ of the adjacent grids decreased.

### 3.3. Analysis of the Different Support Densities

**Figure 14:**
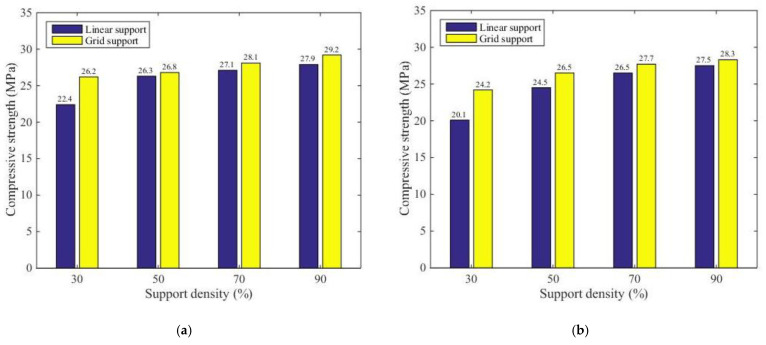

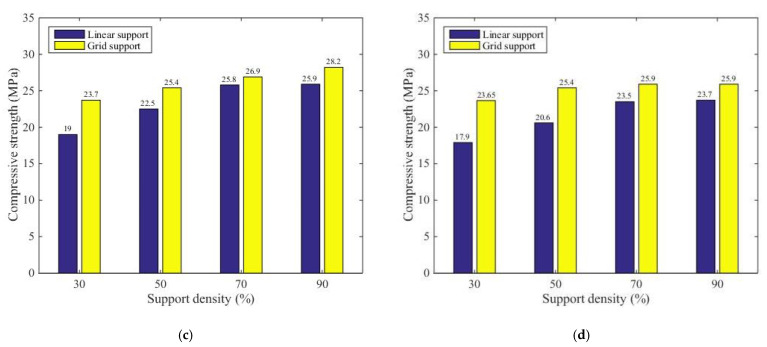
Compressive strength of the two support models with the same Z directional support distance model and different support densities: (**a**) support and model surface and distance Z = 0.08; (**b**) support and model surface and distance Z = 0.10; (**c**) support and model surface and distance Z = 0.12; (**d**) support and model surface and distance Z = 0.14.

Using the data in Table 2 and Table 4 for comparative analysis, Figure 14 plots the average compressive strength values of the components with different support densities and support models, but with the same distance from the support to the model in the Z direction. Here, a is z = 0.08, b is z = 0.10, c is z = 0.12, and d is z = 0.14.

Figure 14 shows that when the distance from the support to the model in the Z direction was the same, with an increase in the support density, the compressive strength of the supported part of the printing component was enhanced under the conditions of both linear support and grid support. However, under the same Z directional support distance model and the same support model, the growth rate of the compressive strength decreased with an increase in the support density. With the same support density, the compressive strength of the supported part of the printing component with grid support was larger than that with linear support.

### 3.4. Analysis of the Different Support Distance

**Figure 15:**
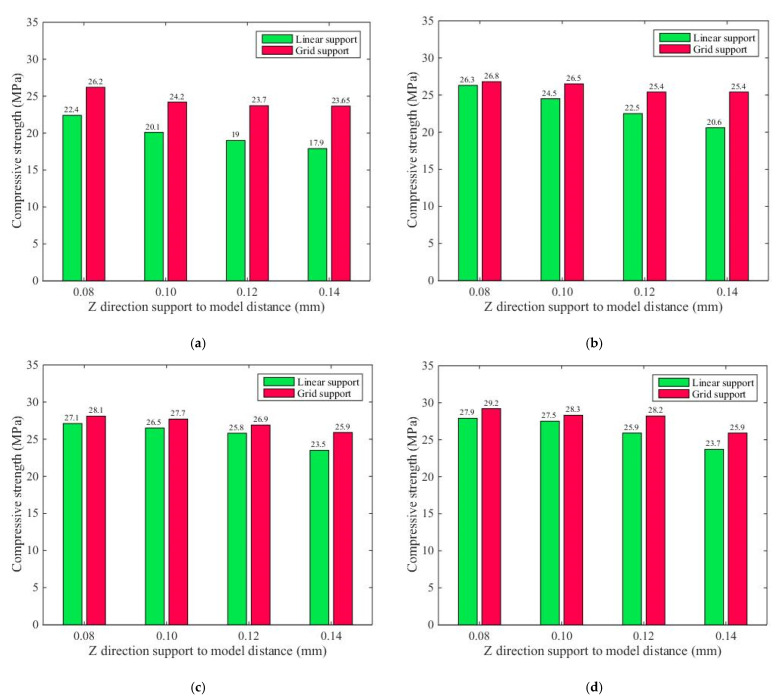
Comparative analysis of the experimental results for the compressive strengths of the two support models with the same support density and different support distance models in the Z direction: (**a**) the supported member printing 30% of the support density of the foundation; (**b**) the supported member printing 50% of the support density of the foundation; (**c**) the supported member printing 70% of the support density of the foundation; (**d**) the supported member printing 90% of the support density of the foundation.

Using the data in Table 2 and Table 4 for comparative analysis, Figure 15 illustrates the average compressive strength of the printing components under both support models with the same support density but different distances from the support to the model in the Z direction. The support density of the printing base of the supported component in Figure 15a was 30%, the support density of the supported component in Figure 15b was 50%, the support density of the supported component in Figure 15c was 70%, and the support density of the supported component in Figure 15d was 90%.

Figure 15 indicates that when the distance from the support to the model in the Z direction was the same, with the same support density, the compressive strength of the supported part of the printing component with grid support was larger than that with linear support. Figure 15a shows that when the support density was 30% under both support conditions, the compressive strength of the supported part of the printing component was notably larger than that when the support density was 50%, 70%, or 90%. When the distance for the support distance model of the supported part of the printing component increased, the compressive intensity of the supported part of the printing components in both support models decreased gradually.

### 3.5. Component Failure Analysis

**Figure 16:**
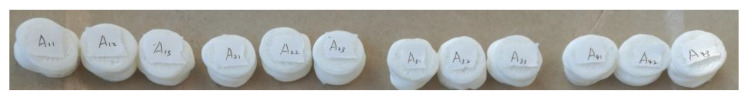
Four sets of experimental parts after compression.

**Figure 17:**
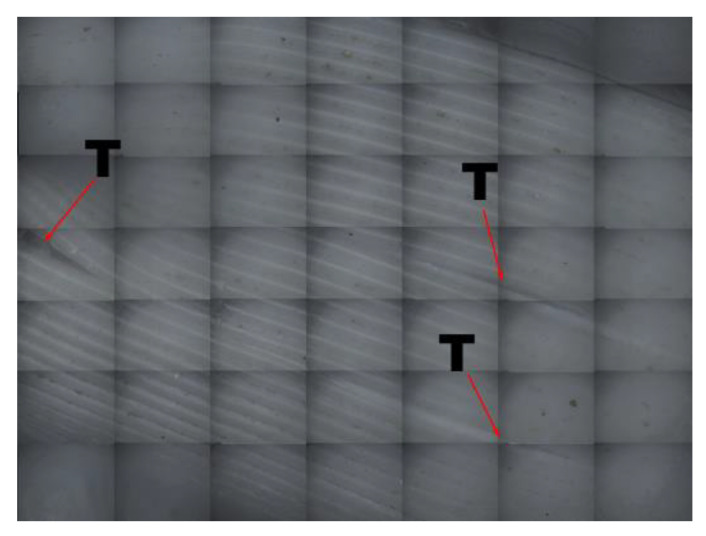
Local distortion and cracking diagram of the grating after component compression.

**Figure 18:**
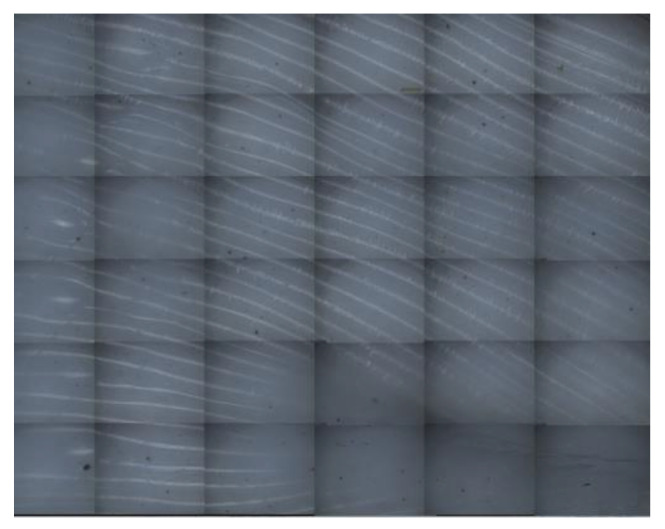
Compressed raster distortion map.

Based on an analysis of the stress diagram of the printing component in the compressive test, the component showed obvious plastic deformation. The compressed samples under the condition of linear support are shown in Figure 16 (where a is Z = 0.08, the shape after the compressive test of the four different linear support density printing components). Here, the cylinder-shaped sample was compressed into a drum shape or pie shape. The adjacent grid layer was observed using a three-dimensional topographic imager (Zeta-20), with cracks appearing between the local adjacent grids, as shown in Figure 17 (location T is a crack in the adjacent grid layer). Moreover, the local fiber was distorted under the press, as shown in Figure 18. Heavily distorted deformation caused cracks between the layers and even local ruptures. However, due to the intense bonding formed by the grid overlap and the even smoothness of the surface grids (with the same filling density and the same distance as the support distance model in the Z direction), the outer cracks on the undersurface of the supporting part and the fiber distortion of the grid support were slightly less severe than those of the supported part of the linear support; the supported part of the linear support also featured slightly less significant deformation than the component without support.

## 4. Conclusions

In this work, 99 groups of compression tests were carried out on FDM-supported positions, and the experimental results were observed and analyzed with a 3D topographic imager. When using models with the same supports, and when the supporting sections were supporting the same distances in the Z direction, with an increase in supporting density, the surface grids of the supported part of the printing component became even and smooth, intense bonding was formed between the adjacent gratings, and the compressive strength increased. However, as the support density increased, the compressive strength of the printing component also slowly increased. When using models with the same supporting densities, the more the supporting distance of the supporting part increased in the Z direction, the lower the compressive strength of the printing component became. The undersurface of the printing component without support did not experience secondary fusion during the printing process. Moreover, the average surface roughness was low, so the undersurface was smooth. When the linear support density was constant, with an increase in the support distance of the supported component in the Z direction, the secondary fusion phenomenon weakened, and the compressive strength gradually increased, with the support density of the printing component being ≥30% and the support distance in the Z direction being ≤0.10, ultimately exceeding the compressive strength of the unsupported member. In contrast, the compressive strength of the grid support model was greater than that of the linear support model and also greater than that of the model without printing support. The present study will help operators select appropriate printing parameters when printing complex components. It is advisable to select an appropriate support model, support density, and distance for the supporting part in the Z direction based on the permissible stress of the component in order to enhance the compressive strength and service life of the printing component. Future studies will include bending tests, fatigue tests, and tensile tests, which will help us further explore these different mechanical characteristics.

## Figures and Tables

**Table 1:** 3D printing—list of other fixed parameters.

Fixed Factors			Control Factors		
Factor	Value	Unit	Factor	Value	Unit
Layer thickness	0.2	mm	Linear support density	30%, 50%, 70%, 90%	
Wall thickness	1.6	mm	Linear support distance in the Z direction	0.08, 0.10, 0.12, 0.14	mm
Fill density	30%		Grid support density	30%, 50%, 70%, 90%	
Nozzle temperature	205	°C	Grid support distance in the Z direction	0.08, 0.10, 0.12, 0.14	mm
Printing speed	50	mm/s			

**Table 2:** Average compressive strength (MPa) of experimental parts with different influencing factors under linear support conditions.

Linear Support Density (%)	Z Direction Support-to-Model Distance (mm)				
	0.08	0.10	0.12	0.14	0
30%	22.40	20.10	19.00	17.90	
50%	26.30	24.50	22.50	20.60	
70%	27.10	26.50	25.80	23.50	
90%	27.90	27.50	25.90	23.70	
0%					20.20

**Table 3:** Measurement results for two adjacent gratings of unsupported printing members and printing members with linear support under different conditions.

Linear Support Density (%)	Z Direction Support-to-Model Distance (mm)		
	0.08	0.14	0.00
70%	$\bar{R_{a}}=168\mu m$ $\bar{X}=773\mu m$	$\bar{R_{a}}=167\mu m$ $\bar{X}=729\mu m$	
90%	$\bar{R_{a}}=164.5\mu m$ $\bar{X}=866\mu m$	$\bar{R_{a}}=145\mu m$ $\bar{X}=848\mu m$	
0%			$\bar{R_{a}}=22.7\mu m$ $\bar{X}=1332\mu m$

**Table 4:** Average compressive strength (MPa) of experimental components with different influencing factors under grid-support conditions.

Grid Support Density (%)	Z Direction Support-to-Model Distance (mm)				
	0.08	0.10	0.12	0.14	0
30%	26.20	24.20	23.70	23.65	
50%	26.80	26.50	25.40	25.40	
70%	28.10	27.70	26.90	25.90	
90%	29.20	28.30	28.20	25.90	
0%					20.20

**Table 5:** Measurement results for two adjacent gratings of unsupported printing members and printing members with grid support under different conditions.

Grid Support Density (%)	Z Direction Support-to-Model Distance (mm)		
	0.08	0.14	0.00
70%	$\bar{R_{a}}=178\mu m$ $\bar{X}=882\mu m$	$\bar{R_{a}}=177\mu m$ $\bar{X}=848\mu m$	
90%	$\bar{R_{a}}=139.5\mu m$ $\bar{X}=1079\mu m$	$\bar{R_{a}}=86.5\mu m$ $\bar{X}=899.6\mu m$	
0%			$\bar{R_{a}}=22.7\mu m$ $\bar{X}=1332\mu m$

## Data Availability

All data are contained within the article.
